# Leakage-Controlled Deep Learning for Multi-Analyte Classification and Quantification in Fast-Scan Cyclic Voltammetry

**DOI:** 10.3390/s26217059

**Published:** 2026-11-08

**Authors:** Navoda Udawaththa, Parth Ganeriwala, Khan Mohammad Al Farabi, Brianna Romero, Siddhartha Bhattacharyya, Pavithra Pathirathna

**Affiliations:** 1 Department of Chemistry and Chemical Engineering, Florida Institute of Technology, Melbourne, FL 32901, USA; nudawaththar2024@my.fit.edu (N.U.); bromero2024@my.fit.edu (B.R.); 2 Department of Electrical Engineering and Computer Science, Florida Institute of Technology, Melbourne, FL 32901, USA; pganeriwala2022@my.fit.edu (P.G.); kfarabi@fit.edu (K.M.A.F.); sbhattacharyya@fit.edu (S.B.)

**Keywords:** neurotransmitters, heavy metals, fast scan cyclic voltammetry, convolutional neural network, artificial neural network

## Abstract

Fast-scan cyclic voltammetry (FSCV) is a widely used electrochemical technique for real-time monitoring of neurotransmitters; however, quantitative interpretation remains dependent on calibration curves generated in simplified in vitro environments that may not accurately reflect the complexity of biological matrices. To address the need for automated FSCV interpretation, we developed a two-stage artificial intelligence (AI) framework integrating analyte classification and concentration prediction. FSCV data were collected for serotonin (5-HT), dopamine (DA), ascorbic acid (AA), and copper (Cu^2+^) using analyte-specific waveforms, generating a dataset of 30,811 cyclic voltammograms from 192 independent experimental runs. Classification models were evaluated using strict run-level partitioning, while regression performance was additionally assessed using run-level and leave-one-electrode-out (LOEO) validation. Four classification architectures were evaluated: a lightweight image-based convolutional neural network (CNN), a feature-engineered multilayer perceptron (MLP), a transfer-learning ResNet18 model, and a one-dimensional CNN (1D-CNN) trained directly on raw voltammograms. The 1D-CNN achieved the highest classification accuracy (99.8%), outperforming transfer learning (98.68%), the lightweight CNN (97.38%), and the feature-based MLP (76.3 ± 0.6%) while maintaining substantially lower model complexity than ResNet18. Input-channel ablation indicated that waveform-specific acquisition protocols contributed to classification performance. For quantitative analysis, analyte-specific artificial neural networks (ANNs) were trained to predict concentrations from voltammetric morphology, achieving coefficients of determination (R^2^) of 0.9958, 0.9970, 0.9849, and 0.9561 for 5-HT, DA, Cu^2+^, and AA, respectively, under the original random sample-level partitioning. However, mean R^2^ values decreased to 0.74–0.88 under run-level validation and 0.61–0.86 under LOEO validation, with conventional peak-current calibration outperforming the ANNs under LOEO conditions. Integration of classification and regression models into an automated inference pipeline enabled sequential analyte identification and concentration estimation from FSCV data. In an additional end-to-end evaluation using the 1D-CNN classifier and analyte-specific ANN regressors, incorrect analyte routing, although infrequent (approximately 0.8%), produced substantial concentration-prediction errors. These results demonstrate the potential of morphology-aware deep learning for automated FSCV interpretation while highlighting the need for improved quantitative reliability and further validation using independent electrodes, mixed-analyte samples, and complex biological matrices.

## 1. Introduction

Neurodegenerative disorders such as Parkinson’s disease arise from multifactorial etiologies involving dysregulation of neurotransmitter systems in combination with environmental stressors, including exposure to toxic metal ions. Understanding this complex interplay requires analytical platforms capable of monitoring rapid neurotransmitter fluctuations while simultaneously distinguishing chemically similar or coexisting species within physiologically relevant matrices. Traditional analytical techniques for neurotransmitters and metal ions, including chromatography-based assays and spectroscopic methods, often rely on postmortem samples or offline analysis, limiting their ability to capture real-time neurochemical dynamics [1,2].

Fast-scan cyclic voltammetry (FSCV) has emerged as a powerful electrochemical technique for sub-second detection of electroactive neurotransmitters in vivo. FSCV is most commonly implemented using carbon-fiber microelectrodes (CFMs), which provide a small geometric area, high temporal resolution, biocompatibility, low background currents, and favorable adsorption kinetics [1,2,3]. These characteristics enable sensitive monitoring of rapid concentration changes in species such as dopamine (DA) and serotonin (5-HT) within complex biological environments. Despite its advantages, FSCV quantification commonly relies on calibration curves generated in vitro. Because electrodes cannot be calibrated directly within brain tissue, calibration is typically performed using electrodes in artificial cerebrospinal fluid or buffer systems designed to approximate physiological conditions. During in vivo experiments, measured signals are interpreted using these pre-established calibration relationships. Although pre- and post-experimental calibrations are conducted to account for electrode surface changes, discrepancies between simplified in vitro matrices and the heterogeneous brain microenvironment introduce uncertainty in quantitative interpretation. Variations in pH, ionic strength, protein adsorption, biofouling, and interfering electroactive species challenge the translatability of conventional calibration-based approaches [3,4]. In addition to conventional calibration approaches, modifications to FSCV scanning waveforms have been investigated to improve electrochemical signal acquisition and processing. For example, Cherkassova et al. proposed a paired-triangular waveform consisting of two consecutive potential scans, with subtraction of the corresponding voltammograms to obtain a differential signal associated with DA [5]. Consequently, there is a growing need for analytical frameworks that can account for complex signal variations without relying solely on predefined in vitro calibration models. In this context, artificial intelligence (AI)-based techniques offer a promising alternative.

AI provides a data-driven alternative by learning morphology–concentration relationships directly from voltammetric signals, rather than relying exclusively on manually extracted peak descriptors and linear calibration curves. In electrochemical signal analysis, two primary neural network paradigms have gained attention: convolutional neural networks (CNNs) [6] and artificial neural networks (ANNs) [7]. CNNs are particularly well suited for structured signals such as voltammograms because convolutional layers learn localized morphological motifs, such as peak shapes, curvature transitions, and slope variations, that combine into higher-level representations. CNNs can operate either on image-rendered voltammograms or directly on raw numerical waveforms using one-dimensional convolutions [8,9]. By learning hierarchical waveform features, CNNs can reduce reliance on manual peak selection and capture discriminative patterns across voltammetric responses. ANNs, in contrast, are general nonlinear function approximators [7,10] that map fixed-length input vectors to continuous outputs. In electrochemical applications, ANNs are commonly employed for regression tasks, where the objective is to predict analyte concentration from waveform features. When trained with appropriate regularization and validation strategies, ANNs can capture nonlinear concentration-dependent distortions that extend beyond simple peak current scaling, enabling more flexible quantitative modeling.

Several studies have explored AI-assisted FSCV analysis. Matsushita et al. applied CNNs to color plots for DA detection [11]. Choi et al. compared one-dimensional CNN (1D-CNN) architectures with principal component regression [12,13] to differentiate structurally similar catecholamines and indoleamines [14]. The Hashemi group employed ANNs to quantify 5-HT using fast-scan controlled-adsorption voltammetry, a variant of FSCV capable of measuring basal concentrations, with the aim of reducing reliance on conventional calibration procedures [15]. Although these investigations demonstrate the promise of deep learning for electrochemical interpretation, important challenges remain, particularly regarding data leakage, run-level generalization, and integrated analyte identification and concentration prediction [16,17]. A critical methodological concern in electrochemical datasets is leakage arising from replicate scans within the same experimental run [18,19]. If scans from a single run are distributed across training and test partitions, models may inadvertently learn run-specific artifacts rather than analyte-generalizable morphology, resulting in inflated performance estimates. Rigorous run-level partitioning is therefore essential for assessing generalization to unseen experimental runs [19].

Here, we present a two-stage deep learning framework specifically designed for analyte identification and quantitative concentration prediction using FSCV measurements of four separately investigated analytes under analyte-specific potential waveforms. In the first stage, a 1D-CNN [8,9] is trained on normalized cyclic voltammograms (CVs) to classify multiple analytes, including 5-HT, DA, ascorbic acid (AA), and copper (Cu^2+^). This model is benchmarked against alternative architectures, including a lightweight image-based CNN, a feature-engineered multilayer perceptron (MLP) [7], and a transfer-learning-based ResNet model [20,21,22], to evaluate performance and parameter efficiency across representations. In the second stage, analyte-specific ANNs are trained for concentration regression, providing quantitative estimates conditioned on the predicted analyte identity. This modular design provides classification confidence scores and analyte-specific concentration estimates. Classification models are evaluated using run-level partitioning to prevent voltammograms from the same experimental run from appearing in both training and test datasets. The regression models are additionally assessed using concentration-stratified run-level partitioning and leave-one-electrode-out validation to examine quantitative generalization across experimental runs and independently fabricated electrodes.

The analyte-specific regression models are integrated into an automated end-to-end inference pipeline that processes raw comma-separated values (CSV) files through classification and concentration estimation without manual intervention. The framework supports automated analysis of separately measured analytes, while its performance under mixed-analyte conditions and in complex biological matrices remains to be established. Collectively, this study develops and systematically evaluates an integrated AI-assisted FSCV framework that combines analyte classification with quantitative concentration prediction while explicitly examining the effects of data partitioning and electrode variability on model performance.

## 2. Materials and Methods

### 2.1. Chemicals

Analytical reagents were obtained from Sigma-Aldrich (St. Louis, MO, USA) unless otherwise indicated and were used as received. L-ascorbic acid was purchased from Fisher Chemical (Pittsburgh, PA, USA) for the preparation of AA solutions. Dopamine hydrochloride was purchased from Alfa Aesar (Tewksbury, MA, USA), and serotonin hydrochloride was purchased from Tokyo Chemical Industry Co., Ltd. (Tokyo, Japan). Copper(II) sulfate was purchased from Fisher Scientific (Hampton, NH, USA) as the Cu^2+^ source. All analyte solutions were prepared separately in tris buffer consisting of 15 mM tris hydrochloride, 140 mM NaCl, 3.25 mM KCl, 1.2 mM CaCl_2_, 1.25 mM NaH_2_PO_4_, 1.2 mM MgCl_2_, and 2.0 mM Na_2_SO_4_. The buffer pH was adjusted to 7.4 and measured using a Mettler Toledo F20 pH meter (resolution: 0.01 pH unit).

### 2.2. Electrode Fabrication and Electrochemical Experiments

Single-bore CFMs were constructed using 7 µm-diameter carbon fibers (Goodfellow, Pittsburgh, PA, USA) and borosilicate glass capillaries (1.0 mm outer diameter and 0.58 mm inner diameter; Sutter Instruments, Novato, CA, USA). A single carbon fiber was inserted into each capillary, which was subsequently tapered using a vertical micropipette puller (PE-100, Narishige Group, Tokyo, Japan) to encapsulate the fiber within the glass. The exposed carbon fiber was manually trimmed to approximately 140–150 µm. Electrical contact between the fiber and an external connecting wire was established by introducing a small quantity of mercury into the capillary.

FSCV measurements were conducted using a two-electrode configuration, with a CFM serving as the working electrode and an in-house-fabricated Ag/AgCl electrode as the reference electrode. Data acquisition was performed using the Quad-UEI system (Electronics Design Facility, University of North Carolina, Chapel Hill, NC, USA). For each analyte, three independently fabricated CFMs were used, with four 20 s experimental runs recorded per electrode at each concentration, yielding 12 runs per concentration. Voltammograms were acquired at 10 Hz, corresponding to one scan every 100 ms. Data are presented as the mean of replicate measurements, with error bars indicating the standard error of the mean (SEM), where applicable.

### 2.3. Data Sources and Dataset Organization

Electrochemical measurements were stored as CSV files containing paired potential and current columns when available. For image-based classification, each voltammogram was rendered as an axis-free grayscale PNG image by plotting the trace as a black line on a white background, removing axis ticks and spines, and exporting at 300 dpi with tight bounding boxes. To enable efficient dataset updates, the plotting routine skipped regenerating images when corresponding PNG files already existed.

For raw-curve classification, each CSV trace was converted into a fixed-length representation of 300 samples to enforce consistent tensor dimensionality during training and inference. When both potential (x) and current (y) columns were present, both were used. If only a single column was available, it was treated as the signal, and a synthetic potential axis was generated using linear spacing across the trace length. This procedure maintained a consistent input format for classification.

For image ingestion, the dataset was organized into analyte-specific directories (e.g., training_data/<analyte>/*.png), and labels were inferred directly from folder names. To support run-level grouping, filenames were required to contain a run identifier in the form **…**run<N>**…**. The run key was defined as the analyte folder name concatenated with the filename prefix up to the run index, uniquely identifying each experimental run. After excluding 88 empty PNG files, the image-based dataset comprised 30,811 valid voltammograms.

### 2.4. Data Partitioning Strategy

To prevent data leakage [18,19] during analyte classification, splitting was performed at the run level. Unique run identifiers were stratified by analyte to preserve class proportions. A two-stage split was applied: test runs were separated first, followed by the division of the remaining runs into training and validation partitions. This design ensured that all scans associated with a given experimental run remained within a single partition, thereby evaluating generalization to unseen runs rather than memorization of run-specific noise [19].

For raw-curve, 1D-CNN classification, run identifiers were derived directly from the CSV directory structure, incorporating analyte, electrode folder, concentration folder, and run folder in the structured format {analyte}/{electrode}/{concentration}/{run}. Stratified splitting was then performed over these run-level keys to maintain class balance.

To evaluate generalization across independently fabricated electrodes, leave-one-electrode-out (LOEO) validation was performed using three electrodes per analyte. In each of three folds, all runs from one electrode per analyte were reserved for testing, while the remaining runs were divided into training and validation partitions at the run level. Because separate experimental-session identifiers were unavailable, electrode identity was used as the highest available grouping level.

To examine the influence of analyte-specific waveform protocols on classification, an input-channel ablation analysis compared three 1D-CNN configurations: combined current and potential, current only, and potential only. Each configuration was evaluated using five independent run-level partitions (random seeds 0–4) and three LOEO folds.

### 2.5. Image-Based CNN Classification Models

Three image-based classification approaches were evaluated. The lightweight CNN comprised three convolutional blocks incorporating convolution, batch normalization, rectified linear unit (ReLU) activation, and max pooling [23,24,25,26], with two-dimensional dropout (Dropout2d) regularization. These blocks were followed by adaptive global average pooling [27] and a compact classifier head (64 → 32 → 4). Training employed cross-entropy loss [28,29] with Adam optimization (learning rate 1 × 10^−3^) [30], StepLR scheduling (step size 5, γ = 0.8), and early stopping based on validation accuracy [31]. Images were resized to 64 × 64 pixels and normalized to the [0, 1] range.

To benchmark engineered descriptors, a feature-based MLP [7] was implemented using approximately 51 handcrafted image features, including Hu moments [32], contrast and texture metrics, and edge-density statistics derived from Canny edge detection [33]. Feature vectors were standardized prior to training and optimized using Adam [30] with StepLR scheduling. The MLP contained 54,724 trainable parameters.

Transfer learning [21,22] was performed using a pretrained ResNet18 [20] backbone with frozen convolutional weights and a newly initialized classifier head (input features → 256 → 128 → 4) incorporating dropout [34] regularization. Images were resized and cached at 224 × 224 pixels, and only the classifier head parameters were optimized during training.

### 2.6. Signal Preprocessing for 1D CNN Classification

For 1D-CNN classification [8,9], each voltammogram was interpolated onto a uniform parameter grid spanning 0 to 1 to produce exactly 300 samples independent of the original sampling density. Linear interpolation preserved waveform continuity while enforcing fixed-length inputs. After interpolation, each channel (potential and current) was standardized on a per-trace basis by subtracting the mean and dividing by the standard deviation with a small numerical stabilizer. This preprocessing reduced sensitivity to absolute signal magnitude and emphasized waveform morphology, although analyte-specific waveform protocols could still influence classification. Traces containing fewer than two valid numeric samples were excluded to avoid instability. Regression inputs were processed differently to preserve concentration-dependent current amplitude. No per-trace normalization was applied. Instead, current traces were padded or truncated to their analyte-specific native input lengths: 240 points for serotonin, 900 for ascorbic acid, 950 for Cu^2+^, and 900 for dopamine. Feature-wise standardization was performed using a StandardScaler. For the additional run-level and LOEO evaluations, the scaler was fitted exclusively to the training partition and subsequently applied to validation and test data. In the original random sample-level regression analysis, the scaler was fitted before partitioning, introducing a potential source of information leakage, as discussed in Section 2.8. When both columns were available, the current (y) channel was preferentially used for regression.

### 2.7. Raw-Curve 1D CNN Classification

Raw-curve analyte classification employed a 1D-CNN [8,9] architecture with sequential convolutions (kernel sizes 7, 5, 5, and 3), batch normalization [23], ReLU activations [24,25], max pooling [26], adaptive average pooling [27], and a dropout-regularized [34] linear head (128 → 64 → 4). Cross-entropy loss [28] was used, with optional inverse-frequency class weighting to mitigate imbalance. The network generated class probabilities for 5-HT, DA, AA, and Cu^2+^.

### 2.8. Analyte-Specific ANN Regression Models

Concentration regression was performed using analyte-specific fully connected ANNs [7,10]. Each network accepted an input vector matching the analyte-specific current-trace length described in Section 2.6, followed by hidden layers of 256, 128, 64, and 32 neurons with ReLU activations [24,25] and dropout regularization (0.3, 0.3, 0.2) [34], terminating in a linear output neuron for concentration prediction. Models were trained using mean squared error loss with Adam optimization (learning rate 1 × 10^−3^) [30] and StepLR scheduling (step size 10, γ = 0.8). Early stopping was governed by validation mean absolute error (MAE), and the best-performing epoch was reloaded prior to final test evaluation [31]. The fitted scaler was serialized and reused during inference.

Analyte-specific ANN regression models were initially evaluated using a random split of individual voltammograms after selecting the analyte subset. Inputs were standardized using a fitted StandardScaler and then divided into training, validation, and test sets. Because scans collected during the same experimental run could appear in multiple partitions, and standardization was performed before splitting, this original analysis was susceptible to data leakage and could overestimate generalization performance.

To address these limitations, regression was additionally evaluated using concentration-stratified run-level partitioning, with 10 of 48 experimental runs per analyte held out for testing across ten random seeds. Stratification maintained the representation of each tested concentration in the training data. Generalization across electrodes was further assessed using three LOEO folds, with five random seeds per fold. In both evaluation strategies, the StandardScaler was fitted using training data only.

To investigate the effects of preprocessing and model selection, amplitude-preserving inputs were compared with per-trace-standardized inputs. Two conventional regression baselines were evaluated using matched partitions: (i) linear calibration based on oxidation-peak current, defined as the maximum forward-sweep current within ±0.1 V of the peak potential identified from training data; and (ii) partial least squares (PLS) regression using the full current trace, with the number of components (1–10) selected using validation data.

The original automated inference pipeline used the ANN models trained with the random sample-level split. The additional run-level and LOEO analyses were used to assess quantitative generalization under more independent testing conditions.

### 2.9. Evaluation Metrics

Classification performance was evaluated using overall accuracy, per-class precision, recall, F1-score, and confusion matrices. Training dynamics were monitored through epoch-wise loss and accuracy curves for both training and validation sets to assess convergence behavior and detect overfitting. Regression performance was quantified using MAE, root mean squared error (RMSE), and the coefficient of determination (R^2^) on a held-out test set following restoration of the optimal validation checkpoint. Predicted-versus-true scatter plots were examined to assess bias and heteroscedasticity. For repeated run-level and LOEO evaluations, results were summarized using the mean and standard deviation where applicable. Mean absolute percentage error (MAPE) and concentration recovery were also calculated to assess quantitative performance across the tested concentration ranges.

### 2.10. End-to-End Inference Workflow

An automated two-stage inference workflow was implemented to integrate analyte identification and concentration estimation. In the original image-based pipeline, a new CSV voltammogram was first rendered as an axis-free PNG and processed by the trained CNN to generate analyte probabilities via softmax output, providing both the predicted identity and a relative classification confidence score. The sample was then routed to the corresponding analyte-specific ANN regressor, which performed padding or truncation, normalization using the saved scaler, and concentration estimation in µM.

A separate run-level evaluation examined error propagation in the 1D-CNN-to-ANN workflow by comparing concentration estimates obtained using the true analyte identity with those obtained using the classifier-predicted identity.

Computational processing times were assessed for both the image-based and raw-curve pipelines. These measurements characterized inference latency but did not establish real-time integration with FSCV acquisition hardware. All analytes were measured separately, and neither mixed-analyte identification nor unknown-analyte rejection was implemented in the automated workflow.

## 3. Results & Discussion

### 3.1. Experimental Design, and Data Quality

Despite its advantages for real-time neurochemical monitoring, quantitative interpretation of FSCV signals remains challenging because electrode responses and experimental conditions can influence voltammetric characteristics. Such variability may not be fully captured by conventional calibration approaches [4].

AI-based analysis offers a potential strategy to mitigate these limitations by learning the current–potential relationship directly from FSCV data. Rather than relying exclusively on peak currents and linear calibration relationships [12,13,35], AI models can extract morphological features from the entire CV and infer both analyte identity and concentration from complex signal patterns [16,17]. To evaluate this approach, three neurochemically relevant analytes, 5-HT, DA, and AA, were selected to represent electroactive species commonly encountered in the brain. Cu^2+^ was included as a representative toxic metal ion capable of interfering with neurotransmission and contributing to neurochemical dysregulation [36].

**Figure 1:**
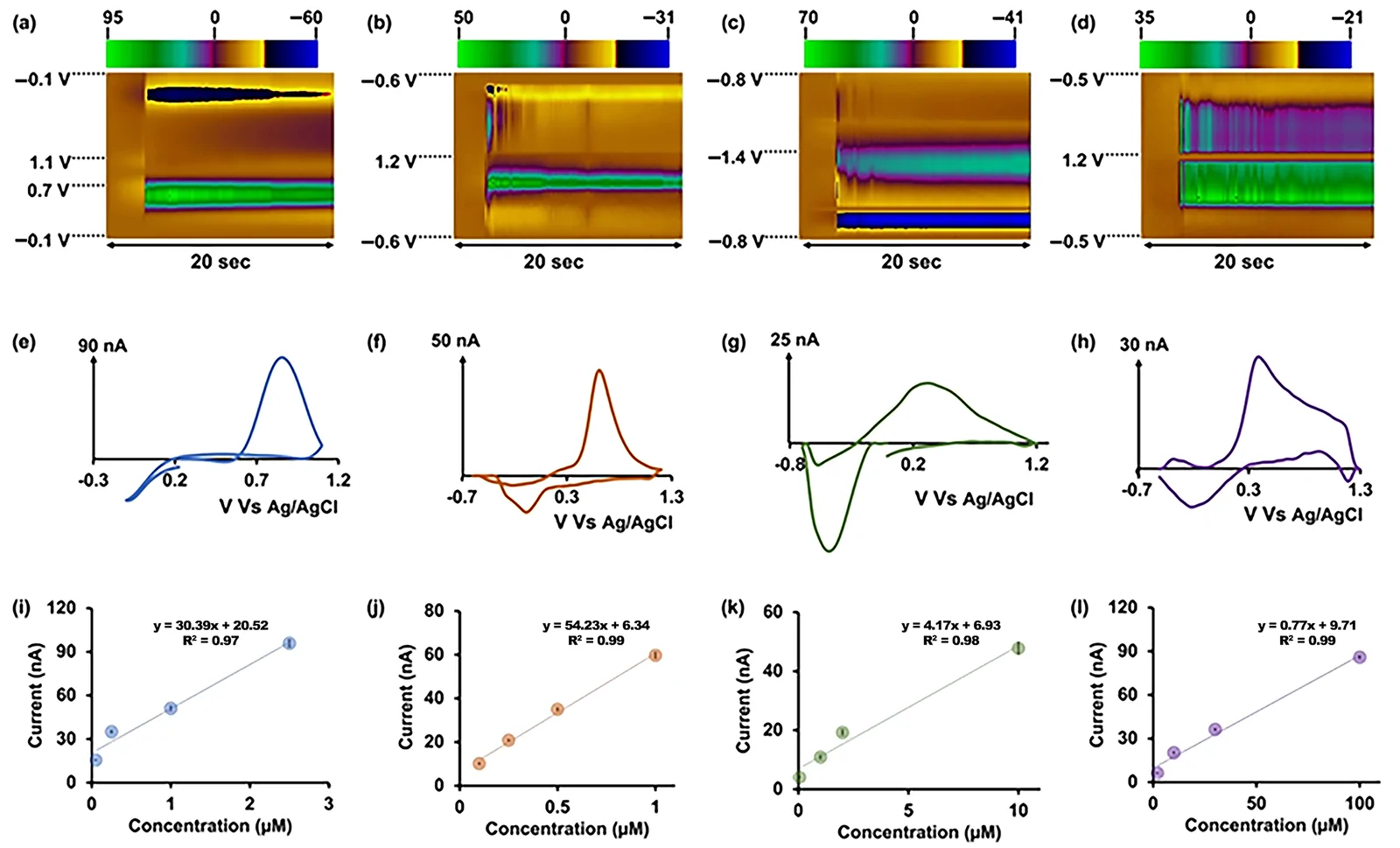
Representative FSCV color plots (**a**–**d**), CVs (**e**–**h**), and corresponding linear calibration curves (**i**–**l**) for 5-HT, DA, Cu^2+^, and AA obtained in tris buffer (pH 7.4) using analyte-specific waveforms.

FSCV data were collected in tris buffer (pH 7.4), containing physiologically relevant concentrations of several major inorganic ions to approximate the pH and ionic environment of artificial cerebrospinal fluid. In contrast to prior studies that applied a single waveform across multiple analytes [14], four distinct FSCV waveforms were employed to obtain analyte-specific electrochemical responses. 5-HT CVs were acquired using a −0.1 V to 1.0 V waveform with a resting potential of 0.2 V at 1000 V s^−1^ (Figure 1a). DA was measured using a −0.6 V to 1.2 V waveform at 400 V s^−1^ (Figure 1b). Cu^2+^ detection employed a −0.7 V to 1.0 V waveform with a resting potential of 0 V at 400 V s^−1^ (Figure 1c), while AA was detected using a −0.5 V to 1.2 V waveform at 400 V s^−1^ (Figure 1d). These waveform-specific protocols were selected to support analyte detection and preserve characteristic redox features [37,38,39]. Under ideal conditions, application of these waveforms yields characteristic CV signatures, as illustrated in Figure 1e–h. Although diagnostic oxidation and reduction peaks were observed in nearly all CVs, a subset exhibited distortions or secondary features potentially associated with baseline drift, capacitive artifacts, or transient irregularities. Because AI models evaluate the full waveform rather than isolated peak metrics, such distortions are analytically meaningful and must be considered during model development. Furthermore, the use of analyte-specific waveforms introduces protocol-dependent information that may influence classification performance, as examined through the input-channel ablation analysis.

For each analyte, four concentrations within the linear region of its respective calibration curve were examined (Figure 1i–l). Each experimental run lasted 20 s in a beaker-based configuration, with analyte introduction occurring at approximately 4 s. Voltammograms acquired from 4 to 20 s were extracted for analysis. For each analyte and concentration, 12 independent runs were performed with three electrodes, with four runs recorded per electrode, resulting in a total of 192 runs across four analytes and four concentrations. CVs were recorded at 10 Hz (one scan every 100 ms), generating a large-scale dataset comprising 30,811 individual CV files stored in CSV format. If analyte injection had occurred precisely at 4 s in every experiment, each run would contain 160 CVs, corresponding to 30,720 CVs across 192 runs. The observed count exceeded this theoretical value by 91 CVs (~0.30%), which may reflect small variations in the extracted acquisition windows. This difference is minor relative to the total dataset size.

Prior to model development, comprehensive quality screening of raw CSV files was conducted (Figure S1). Substantial variability in acquisition density and signal amplitude was observed across analytes, reflecting differences in waveform parameters and sampling resolution. The average number of data points per file was 240 for 5-HT, 900 for AA, 950 for Cu^2+^, and 900 for DA. Signal magnitude and dispersion also differed markedly. The largest current span was observed for 5-HT (−110.30 to 162.86; total span 273.16; standard deviation 20.04), whereas Cu^2+^ exhibited a narrower range (−39.79 to 49.55; total span 89.34; standard deviation 8.60). Automated quality assessment identified isolated high-variability CVs for 5-HT, Cu^2+^, and DA, while AA did not exhibit comparable extreme outliers. These findings demonstrate substantial heterogeneity in the recorded signals and emphasize the importance of analyte-specific preprocessing and rigorous validation. The observed waveform variability also motivates evaluating full-waveform models alongside conventional peak-current methods, rather than assuming that more complex models necessarily provide superior quantitative performance.

### 3.2. Image-Based CNN Modeling

A central methodological challenge when applying AI to high-frequency electrochemical datasets is the prevention of data leakage [18,19]. In the context of repeated FSCV scans, leakage occurs when voltammograms originating from the same experimental run are distributed across training and evaluation partitions. Because scans within a single run share electrode-specific surface states, injection timing characteristics, background drift patterns, and correlated noise structures, a model may inadvertently learn run-dependent artifacts rather than analyte-specific electrochemical morphology. Such leakage can artificially inflate reported performance metrics while substantially compromising true generalization to independent experiments [18]. To reduce this risk, dataset partitioning was performed strictly at the run level [19]. All CVs derived from a given experimental run were assigned exclusively to one of the training, validation, or test partitions. The 192 runs were divided into 120, 32, and 40 runs for training, validation, and testing, respectively. In the image-based representation, this corresponded to 19,252, 5140, and 6419 CV images in the respective subsets. This run-level partitioning allows evaluation of generalization to unseen experimental runs, although electrode- and waveform-related dependencies may remain.

Following partitioning, CVs were rendered as standardized axis-free images for CNN ingestion (Figure S2). This visualization step does not introduce additional experimental measurements; instead, it enforces consistent formatting while suppressing non-signal cues such as axis ticks, labels, borders, and scale indicators. By removing these extraneous visual elements, the model is encouraged to learn directly from voltammogram morphology, including peak position, curvature, slope transitions, and overall waveform structure.Three image-based classification strategies were evaluated on the held-out test split (*n* = 6419 images). A lightweight custom CNN achieved 97.38% test accuracy, whereas a feature-based MLP [7], trained on a 51-dimensional handcrafted descriptor set, achieved 76.3 % accuracy (Table 1).

**Figure 2:**
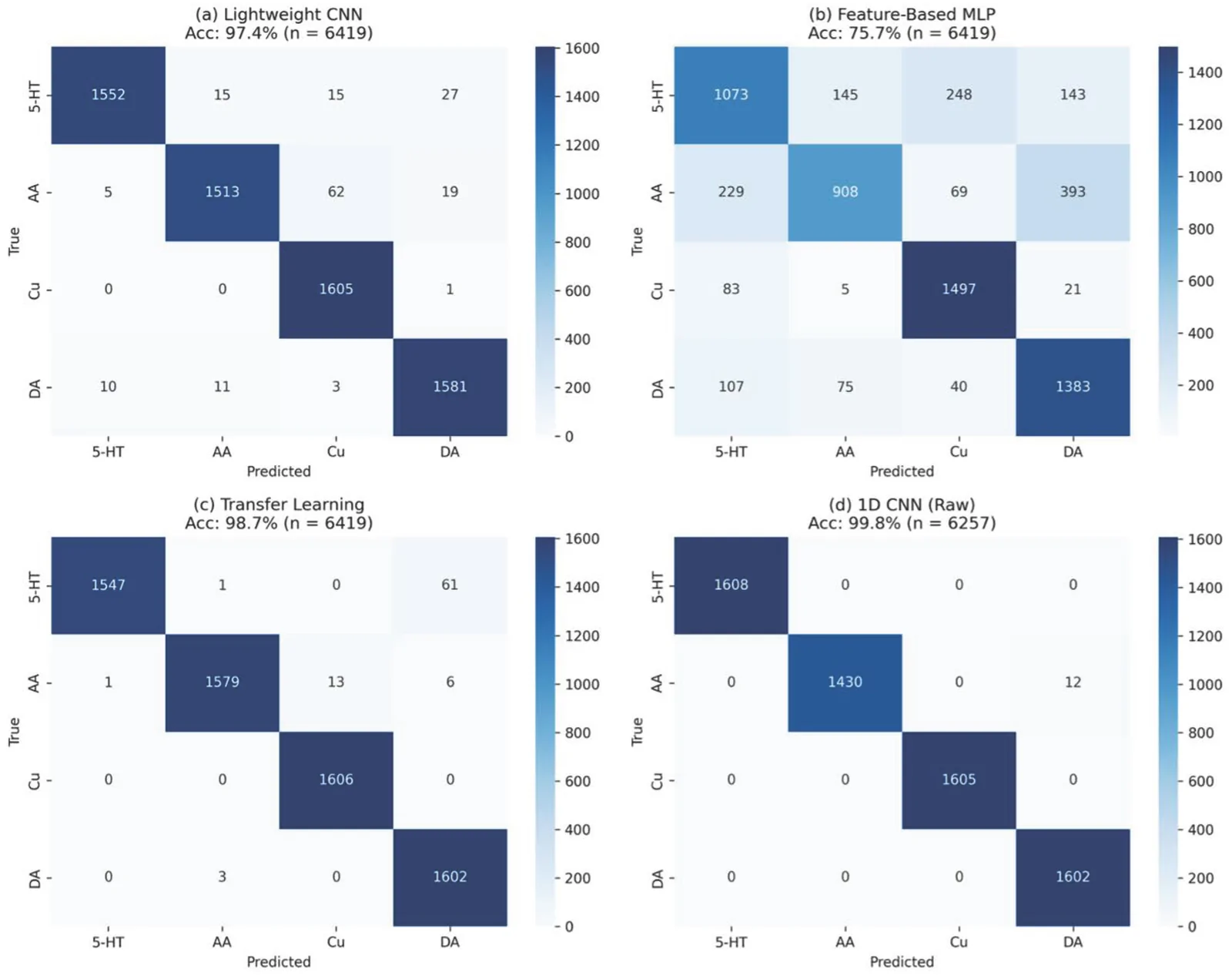
Confusion matrices for the classification of 5-HT, AA, Cu^2+^, and DA using different models on the held-out test sets (*n* = 6419 images for panels a–c; *n* = 6257 curves for panel d): (**a**) lightweight CNN (97.4% accuracy), (**b**) feature-based MLP (75.7% accuracy for training seed 0; 76.3 ± 0.6% across four seeds), (**c**) transfer-learning CNN (98.7% accuracy), and (**d**) 1D-CNN trained on raw data (99.8% accuracy).

The pronounced underperformance of the feature-based MLP suggests that the manually extracted descriptor set did not sufficiently capture the discriminative morphological structure required to separate analytes under realistic run-to-run variability. Consistent with this observation, the macro-averaged F1 score for the MLP was substantially lower than that of either convolutional architecture, indicating weaker performance across the analyte classes. In contrast, the lightweight CNN demonstrated strong per-class performance, achieving high precision across all analytes and a macro-averaged F1 score exceeding 0.97, with reduced recall for AA (94.6%, mainly misclassified as Cu^2+^) and 5-HT (96.5%) relative to Cu^2+^ and DA. This behavior is consistent with the limited cross-class confusions observed in the corresponding confusion matrix (Figure 2).

Transfer learning [21,22] using ResNet18 [20] further improved classification performance, achieving 98.68% test accuracy and a macro-averaged F1 score approaching 0.99, with near-uniform precision and recall across all analytes. However, this incremental improvement was accompanied by a substantial increase in architectural complexity. The ResNet18 model contains 11,341,252 total model parameters, compared to 26,020 parameters in the lightweight CNN. Thus, an absolute gain of approximately 1.3 percentage points in accuracy was associated with an approximately 436-fold increase in total model parameter count. Examination of the confusion matrix indicates that the remaining errors were dominated by 5-HT images classified as DA (61 of 1609 5-HT test images). These results demonstrate that compact, task-specific convolutional architectures can achieve performance approaching that of substantially larger pretrained models for electrochemical image representations while retaining significant efficiency advantages [16,40,41].

From a translational standpoint, the marginal performance improvement afforded by transfer learning must be evaluated in the context of deployability. Large-scale architectures increase memory demand, computational overhead, and inference latency, all of which are critical considerations for integration into portable electrochemical instrumentation or embedded hardware platforms. In contrast, the lightweight CNN achieved near-equivalent classification accuracy with dramatically lower computational burden in terms of model size. For real-time FSCV applications, where rapid inference and potential on-device implementation are desirable, this balance between analytical performance and computational efficiency becomes particularly important. Collectively, these findings suggest that parameter-efficient, task-specific convolutional models may offer a more practical and scalable pathway for AI-assisted electrochemical sensing while accepting a modest reduction in classification accuracy (97.4% versus 98.7% for ResNet18).

### 3.3. Raw-Curve 1D-CNN Classification

Although image-based convolutional models demonstrated strong classification performance, the rendering process inherently introduces an intermediate transformation step in which numerical current–potential data are converted into pixel representations. While this standardization removes non-signal cues such as axis labels and scaling artifacts, it also discretizes continuous electrochemical information into image space. Given that FSCV voltammograms are fundamentally structured current–potential sequences, we next evaluated whether direct sequence-based modeling could further enhance discriminability while preserving the full numerical fidelity of the waveform.

To test this hypothesis, a 1D-CNN [8,9] was trained directly on normalized, fixed-length (300-point) current–potential vectors. This approach eliminates the image-rendering step and allows convolutional filters to operate directly on electrochemical slope transitions, curvature changes, and peak morphologies in their numerical representation. Under the same run-level partitioning constraints [18,19] used for the image-based models, the 1D-CNN achieved 99.81% test accuracy (*n* = 6257 curves; the image-based models used *n* = 6419 images because the image and raw-curve datasets were partitioned independently, so accuracies in Table 1 are not computed on identical samples), with macro-averaged precision, recall, and F1 scores approaching unity. This accuracy represents an individual run-level evaluation and is distinct from the five-seed mean accuracy reported below. The confusion matrix exhibited near-perfect diagonal dominance for 5-HT, Cu^2+^, and DA, with only a small number of residual misclassifications observed for AA, primarily as DA. These errors may reflect similarities in the measured signal shapes, although their precise electrochemical origins cannot be established from the classification results alone.

Because each analyte was recorded with its own waveform, a classifier could in principle identify the waveform protocol rather than the analyte. The 1D-CNN was therefore retrained with both channels, current only, and potential only. Under run-level partitioning (five seeds), the mean test accuracy was 99.7 ± 0.6% for both channels, 99.8 ± 0.1% for current only, and 74.4 ± 0.04% for potential only; under leave-one-electrode-out partitioning (three folds), the corresponding accuracies were 99.95%, 99.6%, and 75.0%, respectively (Table S1). The potential channel alone, which encodes the waveform but not the electrochemical response, classified 5-HT and Cu^2+^ perfectly in every run and fold but could not separate DA from AA, whose waveforms are nearly identical (74.4% overall accuracy, compared with a 25% chance-level baseline for four balanced classes). The waveform alone therefore identifies two of the four analytes, whereas the current-response information enabled discrimination between DA and AA under the evaluated experimental conditions. Because the current trace on the resampled grid still reflects each waveform’s potential window and scan rate, the present data cannot fully separate analyte chemistry from protocol; evaluation on analytes recorded with a common waveform is needed and is noted as a limitation.

To further assess classification reliability, a held-out-analyte evaluation examined the model’s response to analytes excluded from training. Without an explicit rejection mechanism, 8.5–76% of held-out-analyte scans received classification confidence scores above 0.9, demonstrating that unknown analytes could be assigned incorrect labels with high confidence. The evaluated confidence-based rejection procedure rejected 99.4–100% of held-out-analyte scans, with areas under the receiver operating characteristic curve ranging from 0.97 to 0.999 (Table S2). These results highlight the limitations of relying solely on classification confidence and the importance of incorporating and validating an appropriate unknown-analyte rejection mechanism before practical deployment.

Importantly, the 1D-CNN contained 110,372 parameters, substantially fewer than the 11.3 million parameters in ResNet18 [20], while achieving higher test accuracy on its independently partitioned dataset. In parameter-efficiency terms, the sequence-based CNN achieved higher reported accuracy than the transfer-learning model while using approximately 100-fold fewer total parameters. Training and validation curves converged, apart from one transient validation spike at epoch 13 that recovered within a single epoch (Figure 4), indicating stable overall training behavior but not independently establishing the absence of data leakage [18,19]. Collectively, these findings support the effectiveness of direct current–potential modeling for FSCV classification, while acknowledging the influence of waveform-specific acquisition protocols and the limitations of comparing independently partitioned datasets.

### 3.4. Concentration Regression with Analyte-Specific ANNs

Although the 1D-CNN demonstrated the highest classification accuracy, the initial two-stage framework was constructed using the lightweight image-based CNN as the front-end classifier. This configuration facilitated visual inspection of the rendered voltammograms alongside model predictions, although the more accurate 1D-CNN can also serve as the front-end classifier without requiring image rendering. Processing-time measurements further demonstrated the computational advantage of the 1D-CNN pipeline, which required approximately 6 ms per voltammogram on a graphics processing unit (GPU), compared with 245 ms for the image-based pipeline. These indicative measurements suggest that the 1D-CNN approach is better suited to future real-time implementation, although real-time operation has not yet been experimentally validated.

Following classification, quantitative concentration estimation was performed using four independent ANNs [7,10], each trained as an analyte-specific regressor (5-HT, DA, Cu^2+^, and AA). Separate regression architectures were implemented to account for analyte-dependent electrochemical behavior, including differences in adsorption kinetics, redox reversibility, background charging currents, and concentration-dependent signal distortion. By isolating regression tasks, each ANN was optimized to learn the nonlinear mapping between voltammetric morphology and concentration specific to its assigned analyte. In the inference pipeline, the predicted class label from the lightweight CNN directed the corresponding numerical voltammogram to the appropriate ANN regressor. This modular design conditions concentration prediction on the predicted analyte identity, although classification errors may propagate into the regression stage.

**Figure 3:**
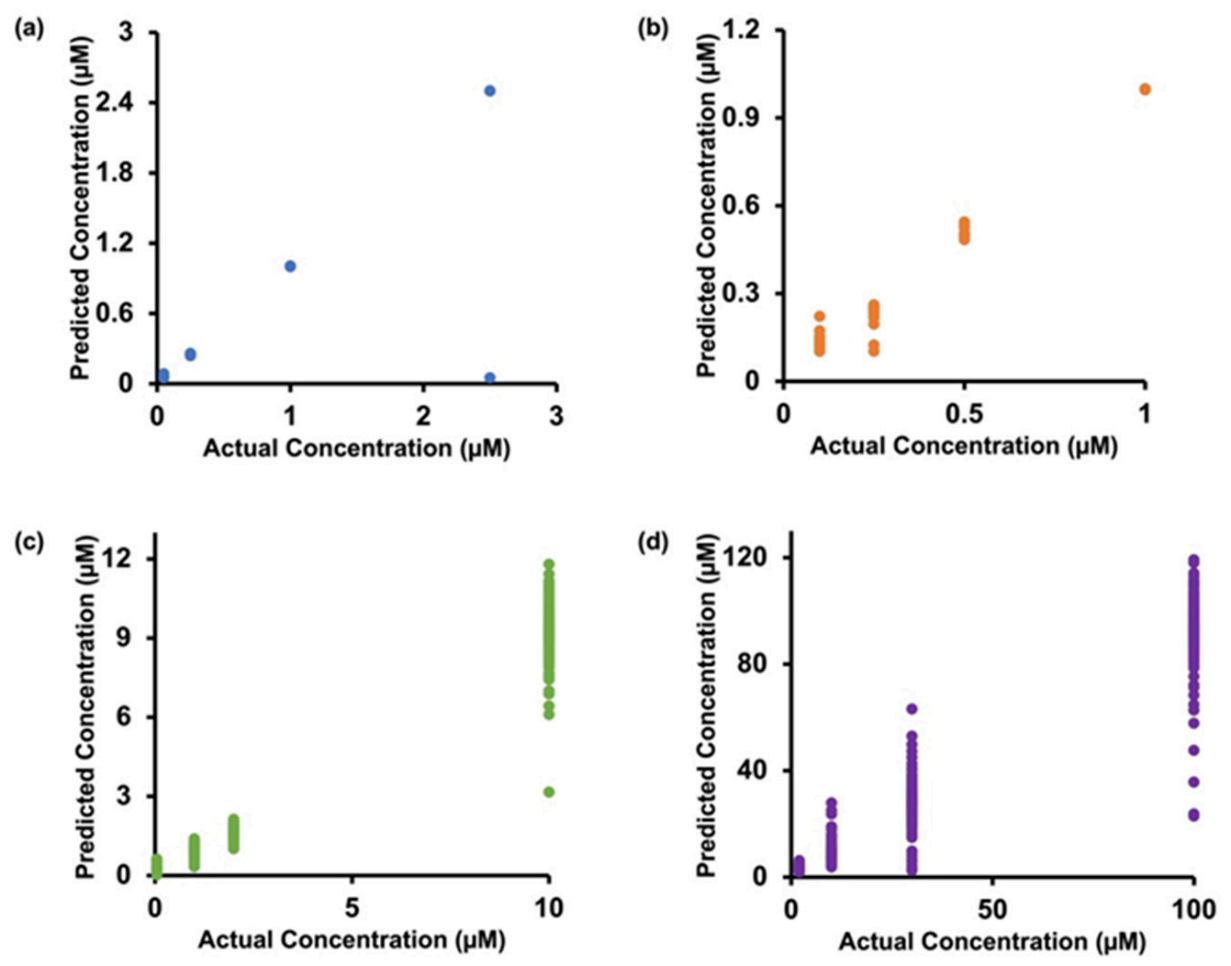
Comparison between actual and predicted concentrations for (**a**) 5-HT, (**b**) DA, (**c**) Cu^2+^, and (**d**) AA using analyte-specific ANNs under the original random sample-level partitioning. The results illustrate performance under this evaluation procedure; independent run-level validation is presented in Figure S3.

Under the original random sample-level partitioning, the analyte-specific ANNs demonstrated high apparent regression performance, with R^2^ values ranging from 0.9561 to 0.9970 across the four analytes (Figure 3). However, because voltammograms from the same experimental run could occur in both training and test sets, these results may overestimate generalization to independent experiments.

To evaluate generalization more rigorously, the regression models were reassessed using run-level and leave-one-electrode-out partitioning. Under run-level validation, mean R^2^ values ranged from 0.74 to 0.88 across analytes, with generally lower performance observed when entire electrodes were excluded from training (Table S3). The variability in individual concentration predictions is illustrated in Figure S3. Under LOEO validation, mean R^2^ values were 0.63 for 5-HT, 0.62 for DA, 0.86 for Cu^2+^, and 0.61 for AA. Substantial overestimation at the lowest calibration levels further revealed limitations in quantitative reliability, particularly near the lower end of the investigated concentration ranges. These findings indicate that the exceptionally high R^2^ values obtained under random sample-level partitioning did not fully translate to independent runs or electrodes.

To assess the analytical value of the ANN approach, its performance was compared with conventional peak-current calibration under LOEO validation (Table S4). Conventional peak-current calibration achieved higher mean R^2^ values than the ANNs for all four analytes: 0.76 versus 0.63 for 5-HT, 0.90 versus 0.62 for DA, 0.94 versus 0.86 for Cu^2+^, and 0.89 versus 0.61 for AA. These results indicate that the ANNs did not outperform conventional peak-current calibration under the evaluated LOEO conditions.

Nevertheless, the ANN approach enables automated concentration estimation from the full voltammogram without requiring an analyst to select a peak-current window. Detailed comparisons of ANN regression performance across validation strategies and against conventional peak-current calibration are provided in Tables S3 and S4. An additional preprocessing sensitivity analysis compared amplitude-preserving ANN inputs with per-trace standardized inputs (Table S5). Preserving current amplitude yielded higher R^2^ values for 5-HT (0.63 versus 0.35), DA (0.62 versus 0.38), and Cu^2+^ (0.86 versus 0.76), whereas the two approaches produced comparable results for AA (0.61 versus 0.63). These findings suggest that current magnitude provides important information for concentration prediction, although concentration-dependent changes in voltammetric morphology may also contribute. Given the variability across validation folds and random seeds, the observed differences should be interpreted cautiously.

Concentration-dependent recovery analysis further revealed limitations in quantitative reliability (Table S6). At the lowest evaluated concentrations, mean recoveries were 283% for 5-HT (0.05 µM), 149% for DA (0.1 µM), 1240% for Cu^2+^ (0.05 µM), and 170% for AA (2 µM). Although recoveries generally approached 100% at higher concentrations, appreciable deviations remained at selected calibration levels. These findings demonstrate that relatively high regression R^2^ values do not necessarily translate into accurate concentration predictions across the entire calibration range.

Taken together, these findings indicate that the lightweight CNN–ANN cascade provides a modular approach to automated analyte classification and concentration estimation, although its quantitative performance depends strongly on the validation strategy [10,13,14]. Morphology-based classification achieved high accuracy under strict run-level partitioning, while analyte-specific nonlinear regression enabled automated concentration prediction with limitations in accuracy, particularly at low concentrations and on unseen electrodes. This modular design supports automated FSCV interpretation but does not establish superior quantification compared with conventional calibration methods.

The proposed regression strategy differs from traditional FSCV quantification approaches that rely primarily on peak-current extraction followed by linear calibration curve fitting [12,13,35]. Unlike conventional peak-current analysis, the ANN regressors use the full current–potential trace to estimate concentration, allowing the models to incorporate waveform features beyond isolated peak amplitudes. However, the present results do not demonstrate improved quantitative accuracy or robustness over conventional calibration. Although external calibration remains necessary for absolute concentration estimation, the proposed framework offers a route toward automated, morphology-aware FSCV analysis. Further validation, particularly using independent electrodes and complex biological matrices, is required before its broader analytical applicability can be established [16,17].

### 3.5. Overfitting and Generalization Analysis

**Figure 4:**
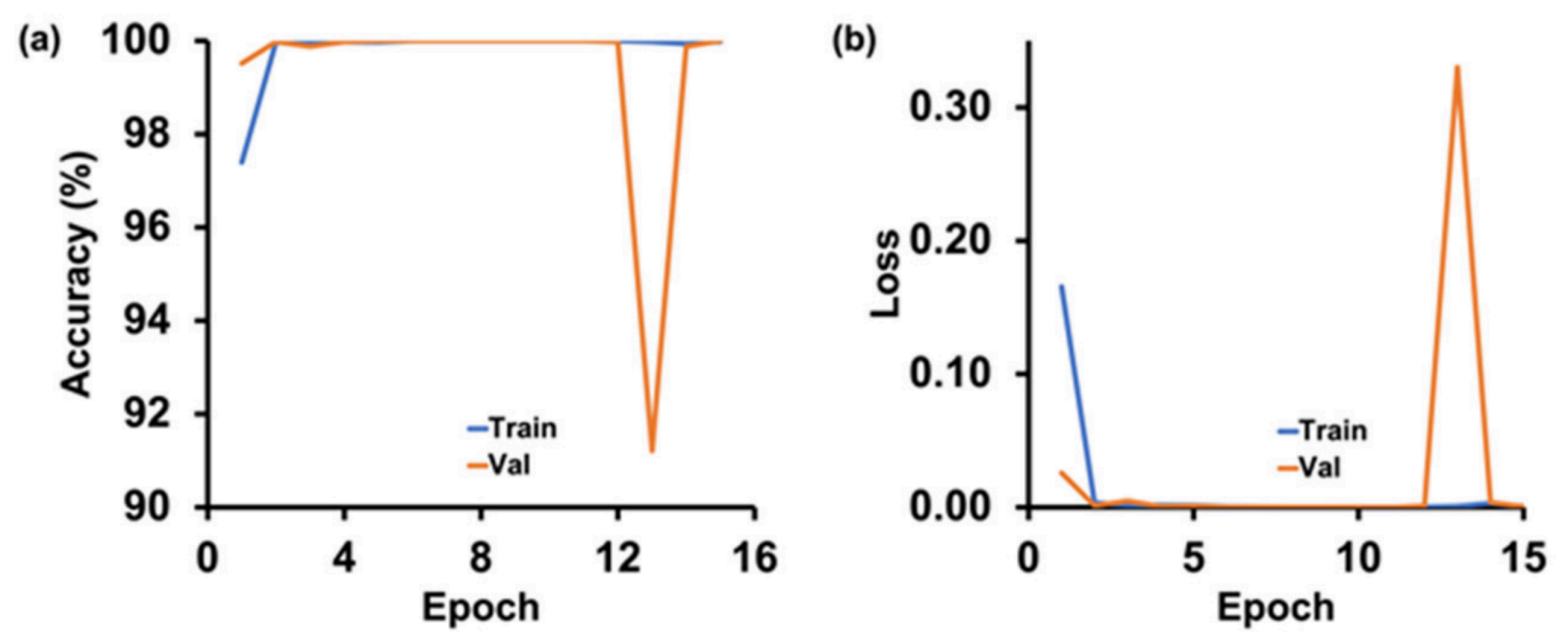
Line plots showing (**a**) training and validation accuracy and (**b**) training and validation loss over 15 epochs for a representative run-level 1D-CNN training experiment for the classification of DA, 5-HT, AA, and Cu^2+^. This training run achieved 100.0% test accuracy; the 99.81% accuracy reported in Section 3.3 was obtained from a separate run-level evaluation.

Generalization performance was systematically evaluated by comparing terminal training and validation metrics and by examining epoch-wise learning dynamics for both classification and regression models. Because all classification experiments were conducted under strict run-level partitioning [18,19], the close agreement between training and validation performance supports stable learning across experimental runs, although it does not exclude the influence of analyte-specific waveform protocols (Section 3.3). For the 1D-CNN classifier [8,9], the training and validation accuracies converged to approximately 100% in the run illustrated in Figure 4, with low terminal losses. A transient decrease in validation accuracy was observed during training, followed by recovery in the subsequent epoch. The overall convergence behavior supports stable optimization, while the independent test results reported in Section 3.3 provide a more direct assessment of classification performance on unseen experimental runs.

**Figure 5:**
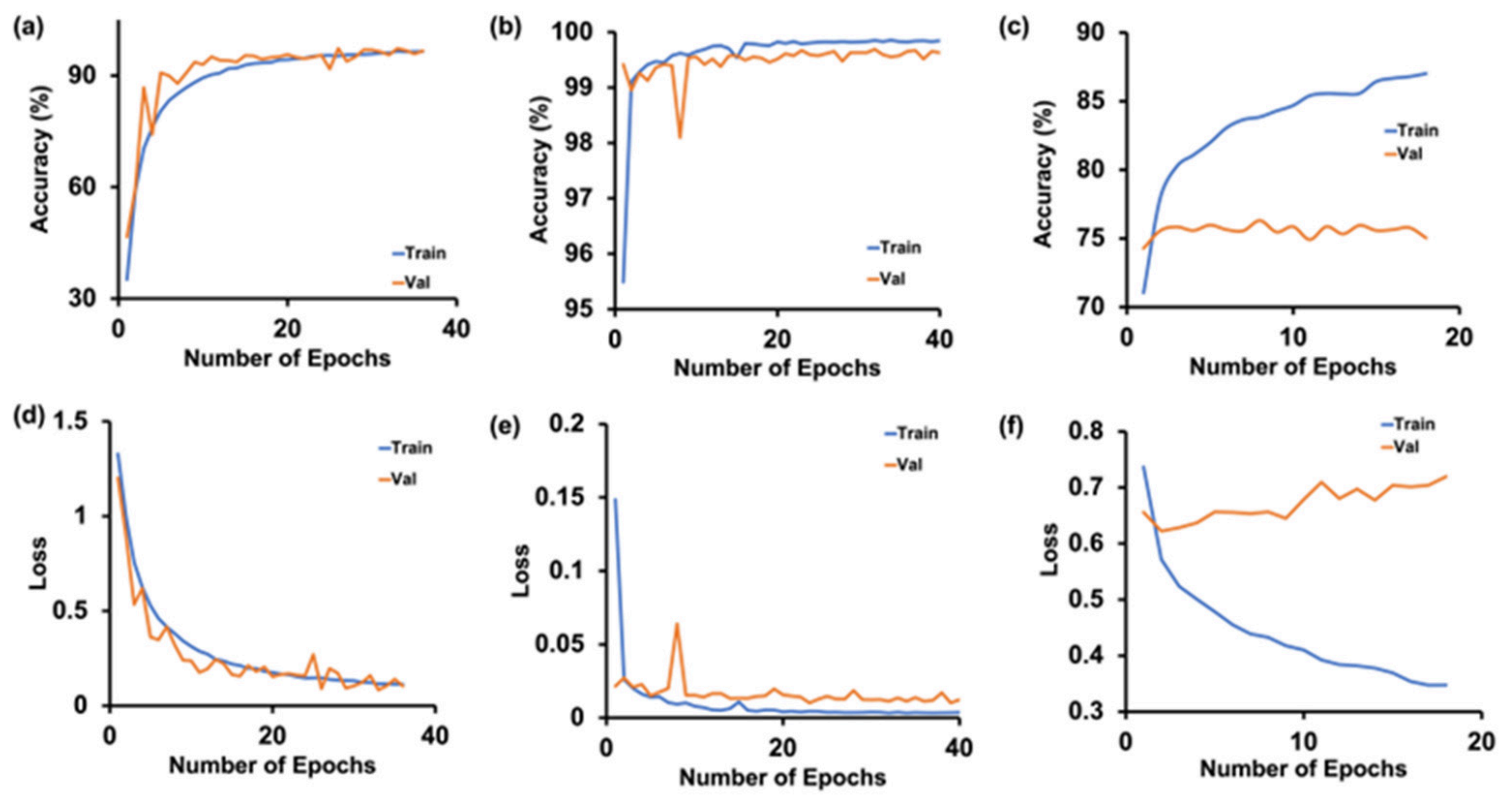
Training and validation performance in terms of (**a**–**c**) classification accuracy and (**d**–**f**) loss for the classification of DA, 5-HT, AA, and Cu^2+^. Results are shown for three different models: (**a**,d) the lightweight CNN, (**b**,**e**) the ResNet18 transfer-learning model, and (**c**,**f**) the feature-based MLP. The convolutional models exhibit relatively close training and validation trajectories, whereas the MLP shows a pronounced train–validation performance gap, consistent with overfitting.

Importantly, under a leakage-prone random split, such performance could be artificially inflated [18]; however, because all scans from a given run were confined to a single partition, these results reflect generalization to unseen experimental runs rather than necessarily to new electrode populations or waveform-independent chemical identification [19]. The lightweight image-based CNN and the transfer-learning ResNet18 [20,21,22] model exhibited similarly stable behavior. Both architectures demonstrated relatively small train–validation accuracy gaps and broadly similar loss trajectories across epochs (Figure 5), consistent with stable convolutional model training under run-level partitioning. In contrast, the feature-based MLP baseline showed a pronounced train–validation discrepancy, consistent with overfitting to the engineered 51-dimensional descriptor space despite substantially lower overall performance. This behavior suggests that the selected handcrafted features provided less effective analyte discrimination than the convolutional representations and were more susceptible to overfitting under the evaluated conditions.

**Figure 6:**
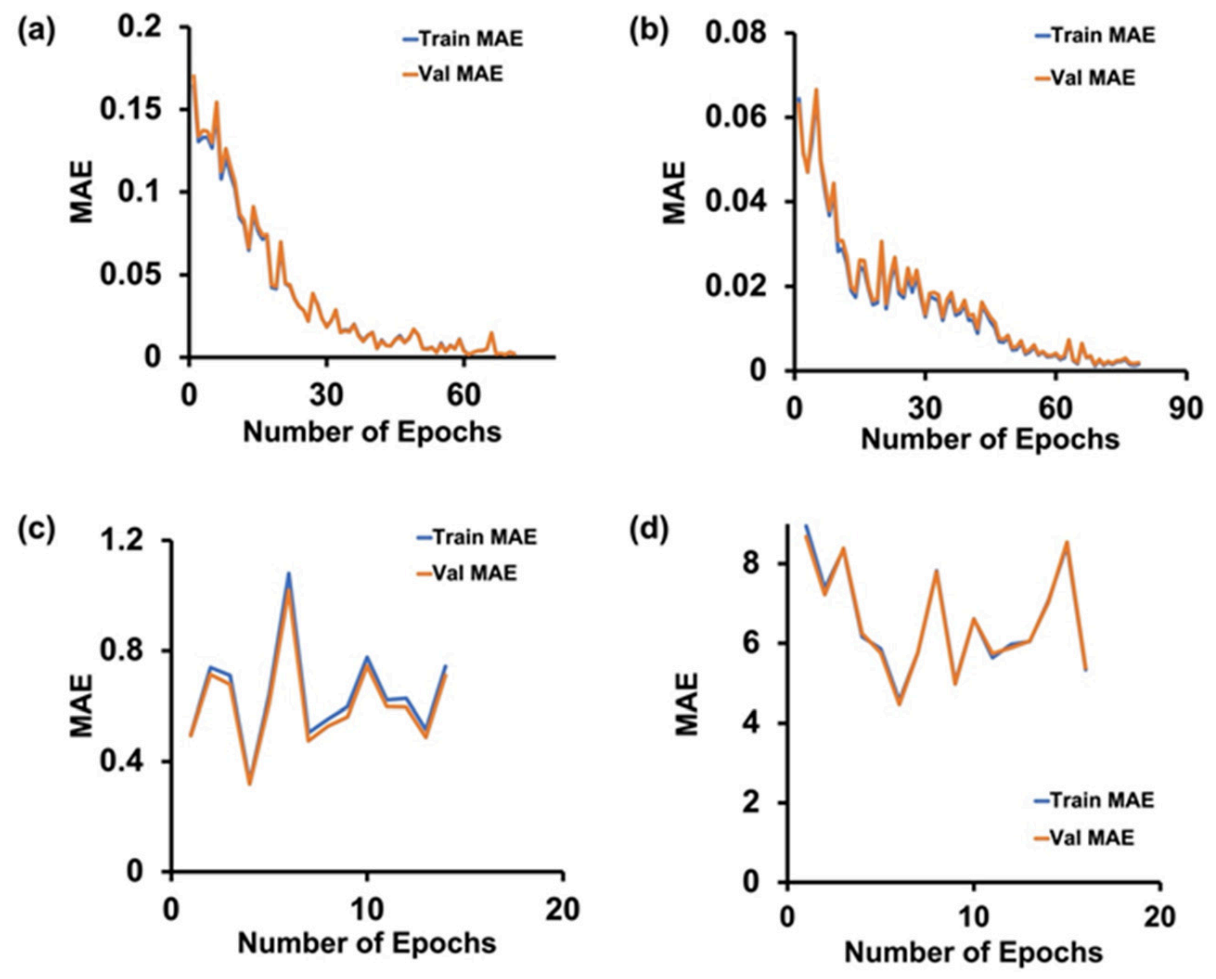
Training and validation MAE over epochs for the prediction of (**a**) 5-HT, (**b**) DA, (**c**) Cu^2+^, and (**d**) AA using the ANN regressors under the original random sample-level partitioning.

Regression models were evaluated using MAE trends across training and validation partitions. The learning curves in Figure 6 were obtained using the original random sample-level partitioning, in which voltammograms from the same experimental run could occur in both training and validation datasets. Consequently, agreement between these curves cannot establish regression generalization to independent runs or electrodes. The 5-HT, DA, and Cu^2+^ ANNs exhibited close alignment between training and validation MAE values, indicating similar performance within the original partitioning scheme (Figure 6). In contrast, the AA regression model displayed a noticeable elevation in validation loss relative to training that may reflect overfitting, greater signal variability, or both. Given AA’s broader dynamic range and greater susceptibility to baseline distortion and peak overlap, this behavior may be associated with analyte-dependent response heterogeneity, although the learning curves alone cannot establish its cause. Overall, the more rigorous run-level and leave-one-electrode-out evaluations presented in Section 3.4 and Table S3 provide a more reliable assessment of regression generalization and reveal limitations that are not apparent from the original learning curves.

To illustrate the inference workflow, a representative scan file (5.0.csv) was processed through the complete CNN–ANN cascade. The lightweight CNN classifier assigned the analyte label 5-HT with 99.14% confidence, and the corresponding analyte-specific ANN predicted a concentration of 0.050104 µM. This end-to-end example illustrates the operational structure of the framework: morphology-based identity assignment followed by conditioned nonlinear regression, yielding both a confidence metric and a quantitative output. However, because the experimental run associated with this file was not documented, the example cannot be considered an independent validation of the deployed pipeline.

To assess end-to-end generalization more rigorously, an additional evaluation combined the 1D-CNN classifier with the analyte-specific ANN regressors using matched run-level partitions across five random seeds. The original dataset comprised 30,811 voltammograms from 192 experimental runs. Across the five partitions, the held-out test sets contained a cumulative total of 31,284 test-scan evaluations, with individual voltammograms potentially appearing in the test sets of multiple partitions. Of these evaluations, 246 (approximately 0.8%) were incorrectly classified and directed to the wrong analyte-specific ANN. Despite this relatively low misclassification rate, incorrect routing substantially affected concentration predictions. For DA, the mean regression R^2^ decreased from 0.62 when the correct analyte identity was supplied to −9.35 when classification-based routing was used. In one partition, just seven incorrectly routed DA scans among 1444 test scans reduced the R^2^ from 0.90 to −48. This finding demonstrates that high classification accuracy does not necessarily guarantee reliable end-to-end quantification, because incorrectly classified voltammograms are directed to regressors trained for different analytes. Importantly, this evaluation used the 1D-CNN front end rather than the lightweight image-based CNN used in the illustrative workflow; therefore, it does not establish the independent end-to-end performance of that image-based configuration. Collectively, the overfitting diagnostics and independent validation results indicate that run-level partitioning [18,19] supports robust convolutional classification of the investigated analytes under their respective experimental protocols. However, the regression models exhibit greater sensitivity to experimental variability, and classification errors can propagate into substantial concentration-prediction errors. The combined framework therefore provides a basis for automated, AI-assisted FSCV analysis, but additional validation and safeguards against incorrect analyte routing are needed before reliable practical deployment can be established [11,14,15,16,40,41].

## 4. Conclusions

In this study, we developed and evaluated a leakage-controlled deep learning framework for automated interpretation of FSCV data, integrating analyte classification with concentration prediction in a unified workflow. By enforcing strict run-level partitioning for classification and subsequently evaluating the regression models under run-level and LOEO partitioning, we reduced the risk of data leakage and assessed model performance on unseen experimental runs and electrodes. Comparative evaluation of four classification architectures demonstrated that convolutional approaches substantially outperformed feature-engineered models, highlighting the importance of learning waveform morphology directly from FSCV data. Among the tested models, the 1D-CNN achieved the highest classification accuracy (99.8%) while maintaining considerably lower complexity than the transfer-learning ResNet18 architecture. However, because each analyte was measured using a distinct acquisition protocol, the classification results may partly reflect waveform-specific information rather than analyte-dependent electrochemical responses alone.

To extend beyond analyte identification, analyte-specific ANN regressors were developed for quantitative concentration estimation. These models exhibited high apparent predictive performance under the original random sample-level partitioning, with R^2^ values ranging from 0.9561 to 0.9970. However, regression performance decreased under independent run-level and LOEO validation, and the ANN models did not outperform conventional peak-current calibration under LOEO conditions. Concentration-dependent recovery analysis also revealed substantial overestimation at the lowest evaluated concentrations. The resulting CNN–ANN framework provides automated analyte classification followed by conditioned concentration prediction, enabling sequential processing of FSCV data within a modular inference pipeline. However, additional end-to-end evaluation using the 1D-CNN classifier demonstrated that even a low misclassification rate (approximately 0.8%) could produce substantial concentration-prediction errors when scans were directed to incorrect analyte-specific regressors.

More broadly, this work demonstrates the potential of AI-driven electrochemical analysis to automate analyte identification and concentration estimation by utilizing information contained in the full voltammogram rather than relying exclusively on isolated peak features. However, the present results do not establish improved quantitative accuracy or robustness over conventional peak-current calibration. Although external calibration remains necessary for absolute concentration scaling, morphology-aware machine learning offers a potentially useful approach to automated FSCV interpretation, provided that its limitations in generalization and quantitative reliability are addressed. The present study was restricted to individual analytes measured in buffer, without evaluating mixed-analyte systems, complex biological matrices, or in vivo conditions. Although preliminary unknown-analyte rejection experiments demonstrated encouraging performance, the current inference pipeline does not incorporate a validated rejection mechanism for unknown or low-confidence inputs. Future studies will focus on extending the framework to mixed-analyte systems, in vivo datasets, and additional neurochemically relevant species, while improving cross-electrode generalization, quantitative accuracy, and safeguards against incorrect analyte routing. These developments will be necessary to advance toward reliable, scalable AI-assisted electrochemical sensing platforms.

## Figures and Tables

**Table 1:** Comparison of model parameter counts and test accuracies for the four classification models evaluated for voltammetric signal classification. The feature-based MLP accuracy represents the mean across four training seeds, whereas the image-based CNN and ResNet18 accuracies correspond to training seed 0. The 1D-CNN was evaluated on a separate held-out test partition.

Model	Reported Parameter #	Test Accuracy
1D-CNN	110,372	99.8%
Light-weight CNN	26,020	97.4%
Feature-based MLP	54,724	76.3%
Transfer-learning ResNet 18	11,341,252	98.7%

## Data Availability

The ANN and CNN models developed and used in this study are available at: https://github.com/ParthGaneriwala/Electrochemical-Detection (accessed on 20 September 2026).

## Supplementary Materials

The following supporting information can be downloaded at: https://www.mdpi.com/article/10.3390/s26217059/s1. Figure S1: Data quality assessment of the FSCV dataset for 5-HT, AA, Cu^2+^, and DA. Metrics include the standard deviation of current responses (a), number of valid CSV files (b), average number of data points per voltammogram (c), and current signal range (d), computed from all valid CSV files used in this study; Figure S2: Representative axis-free CV images generated using a Python-based preprocessing workflow for (a) 5-HT, (b) DA, (c) Cu^2+^, and (d) AA. These standardized PNG representations were used as inputs for the image-based deep learning classification models; Figure S3: Comparison of actual and ANN-predicted concentrations for (a) 5-HT, (b) DA, (c) AA, and (d) Cu^2+^. Each data point represents an individual concentration prediction, and the dashed lines indicate ideal agreement between predicted and actual concentrations (y = x). The scatter illustrates variability in ANN predictions at fixed nominal concentrations, including substantial deviations at lower concentrations. For clarity, four predictions exceeding 4.5 µM in (a) and three predictions exceeding 1.8 µM in (b) are not displayed; Table S1: Classification accuracy of the 1D-CNN model using different combinations of input channels. Run-level results are reported across five random seeds, and leave-one-electrode-out (LOEO) results are reported across three folds. The comparison evaluates the contributions of current-response information and applied-potential information to analyte classification; Table S2: Performance of the evaluated unknown-analyte rejection procedure when each analyte was excluded from the classifier’s training classes. The rejection percentage represents the proportion of excluded-analyte scans identified as unknown, whereas the final column reports the proportion assigned classification confidence above 0.9 in the absence of rejection. These results illustrate the limitations of relying solely on classification confidence for unknown-analyte detection; Table S3: Comparison of analyte-specific ANN regression performance under the original random sample-level partitioning, run-level partitioning, and LOEO validation. Values are expressed as coefficients of determination (R^2^). Run-level and LOEO results are reported as mean ± standard deviation; Table S4: Comparison of ANN-based concentration prediction and conventional peak-current calibration. ANN results are shown under run-level and LOEO validation, whereas peak-current calibration results are reported under LOEO validation. Values are expressed as mean R^2^ ± standard deviation. Direct comparisons between ANN regression and peak-current calibration were therefore made using the corresponding LOEO results; Table S5: Comparison of ANN regression performance using amplitude-preserving inputs and per-trace standardized inputs. Preserving current amplitude improved concentration-prediction performance for 5-HT, DA, and Cu^2+^, whereas AA exhibited comparable performance under the two preprocessing strategies. Values are reported as R^2^; Table S6: Mean recovery of ANN-predicted concentrations at individual calibration levels for 5-HT, DA, Cu^2+^, and AA. Concentrations and corresponding recovery percentages are listed in matching order. The results illustrate concentration-dependent variations in quantitative prediction accuracy, including substantial deviations at the lowest evaluated concentrations; Python code for FSCV data assemble using google colab.
