# Supplementary material for: Leakage-Controlled Deep Learning for Multi-Analyte Classification and Quantification in Fast-Scan Cyclic Voltammetry
Source: Sensors (Basel). 2026 Nov 8;26(21):7059. doi: 10.3390/s26217059 (PMC13766481; doi:10.3390/s26217059)
Supplement: Supplementary file 1 [file sensors-26-07059-s001.zip › sensors-4611779-supplementary.pdf]

Supplementary Information

Table of content:

- Signal variability of FSCV responses
- Axis-free CV images
- ANN concentration prediction performance under independent validation
- Effect of Input Channels on 1D-CNN Classification Performance
- Held-Out-Analyte Rejection Performance
- Effect of Data Partitioning Strategy on ANN Regression Performance
- Comparison of ANN Regression and Conventional Peak-Current Calibration
- Effect of Preprocessing Strategy on ANN Regression Performance
- Concentration-Dependent Recovery of ANN Predictions
- Python code for FSCV data assemble using google colab

Signal variability of FSCV responses

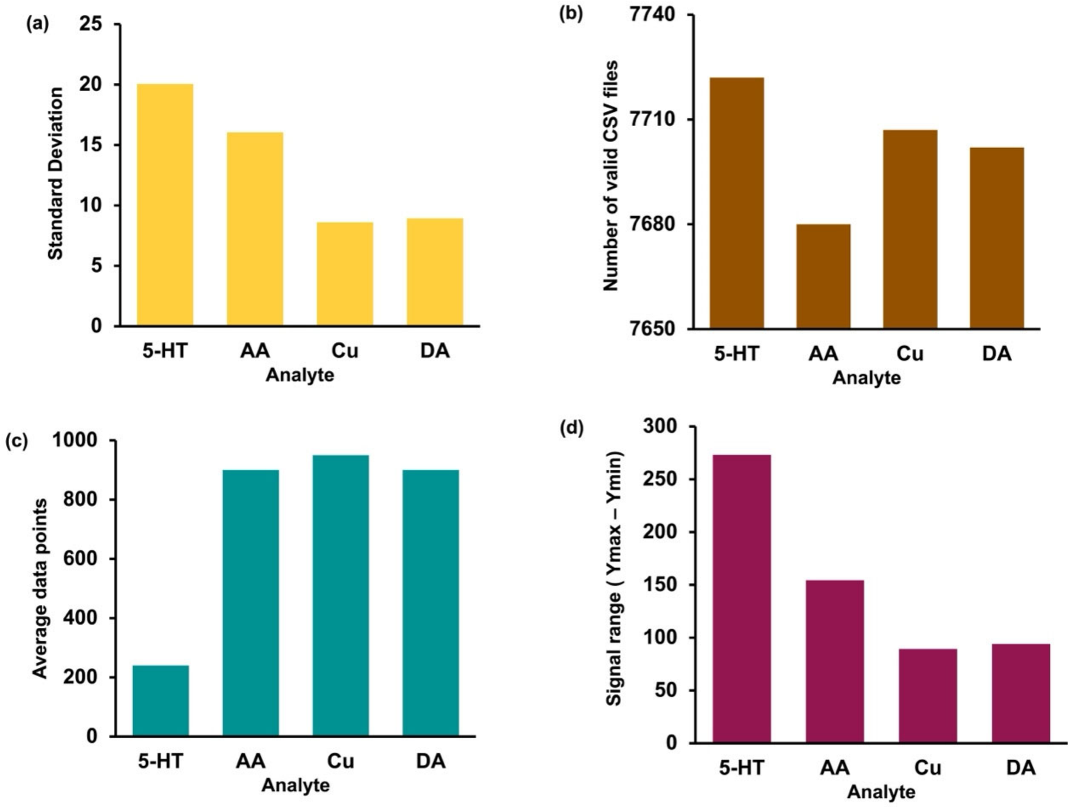

Figure S1. Data quality assessment of the FSCV dataset for 5-HT, AA, Cu^{2+}, and DA. Metrics include the standard deviation of current responses (a), number of valid CSV files (b), average number of data points per voltammogram (c), and current signal range (d), computed from all valid CSV files used in this study.

Axis-free CV images

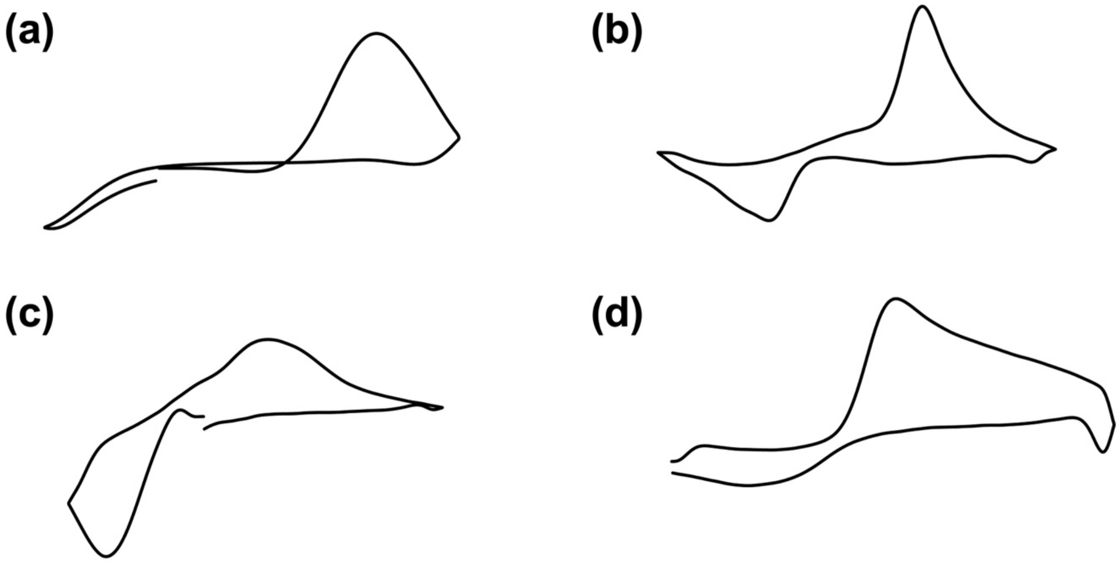

Figure S2. Representative axis-free CV images generated using a Python-based preprocessing workflow for (a) 5-HT, (b) DA, (c) Cu^{2+}, and (d) AA. These standardized PNG representations were used as inputs for the image-based deep learning classification models.

ANN concentration prediction performance under independent validation.

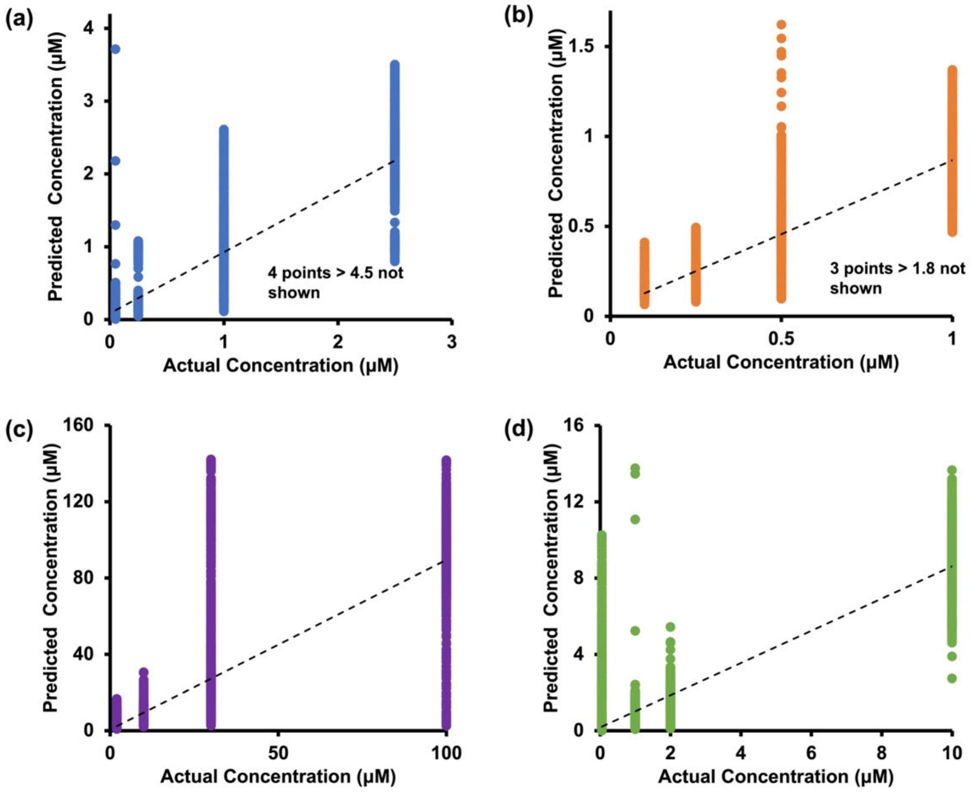

Figure S3. Comparison of actual and ANN-predicted concentrations for (a) 5-HT, (b) DA, (c) AA, and (d) Cu^{2+}. Each data point represents an individual concentration prediction, and the dashed lines indicate ideal agreement between predicted and actual concentrations (y = x). The scatter illustrates variability in ANN predictions at fixed nominal concentrations, including substantial deviations at lower concentrations. For clarity, four predictions exceeding 4.5 μM in (a) and three predictions exceeding 1.8 μM in (b) are not displayed.

Effect of Input Channels on 1D-CNN Classification Performance

Table S1. Classification accuracy of the 1D-CNN model using different combinations of input channels. Run-level results are reported across five random seeds, and leave-one-electrode-out (LOEO) results are reported across three folds. The comparison evaluates the contributions of current-response information and applied-potential information to analyte classification.

1D-CNN input	Run-level accuracy (5 seeds)	LOEO accuracy (3 folds)
Current and potential	99.7 ± 0.6%	99.95%
Current only	99.8 ± 0.1%	99.6%
Potential only	74.4 ± 0.04%	75.0%

Held-Out-Analyte Rejection Performance

Table S2. Performance of the evaluated unknown-analyte rejection procedure when each analyte was excluded from the classifier's training classes. The rejection percentage represents the proportion of excluded-analyte scans identified as unknown, whereas the final column reports the proportion assigned classification confidence above 0.9 in the absence of rejection. These results illustrate the limitations of relying solely on classification confidence for unknown-analyte detection.

Held-out analyte	Unknown scans rejected	Unknown scans assigned confidence above 0.9 without rejection
5-HT	100%	76%
DA	99.7%	8.5%
Cu^{2+}	99.4%	46%
AA	99.9%	35%

Effect of Data Partitioning Strategy on ANN Regression Performance

Table S3. Comparison of analyte-specific ANN regression performance under the original random sample-level partitioning, run-level partitioning, and LOEO validation. Values are expressed as coefficients of determination (R^2). Run-level and LOEO results are reported as mean ± standard deviation.

Analyte	Original random-split R^2	Run-level R^2 (mean ± SD)	LOEO R^2 (mean ± SD)
5-HT	0.9958	0.74 ± 0.35	0.63 ± 0.26
DA	0.9970	0.81 ± 0.09	0.62 ± 0.21
Cu^{2+}	0.9849	0.88 ± 0.07	0.86 ± 0.11
AA	0.9561	0.84 ± 0.14	0.61 ± 0.27

Comparison of ANN Regression and Conventional Peak-Current Calibration

Table S4. Comparison of ANN-based concentration prediction and conventional peak-current calibration. ANN results are shown under run-level and LOEO validation, whereas peak-current calibration results are reported under LOEO validation. Values are expressed as mean $R^2 \pm$ standard deviation. Direct comparisons between ANN regression and peak-current calibration were therefore made using the corresponding LOEO results.

Analyte	ANN run-level R^2	ANN LOEO R^2	Peak-current calibration LOEO R^2
5-HT	0.74 ± 0.35	0.63 ± 0.26	0.76 ± 0.01
DA	0.81 ± 0.09	0.62 ± 0.21	0.90 ± 0.03
Cu^{2+}	0.88 ± 0.07	0.86 ± 0.11	0.94 ± 0.00
AA	0.84 ± 0.14	0.61 ± 0.27	0.89 ± 0.01

Effect of Preprocessing Strategy on ANN Regression Performance

Table S5. Comparison of ANN regression performance using amplitude-preserving inputs and per-trace standardized inputs. Preserving current amplitude improved concentration-prediction performance for 5-HT, DA, and Cu^{2+}, whereas AA exhibited comparable performance under the two preprocessing strategies. Values are reported as R^2.

**Analyte**	**Amplitude-preserving ANN R^2**	**Per-trace standardized ANN R^2**
5-HT	0.63	0.35
DA	0.62	0.38
Cu^{2+}	0.86	0.76
AA	0.61	0.63

Concentration-Dependent Recovery of ANN Predictions

Table S6. Mean recovery of ANN-predicted concentrations at individual calibration levels for 5-HT, DA, Cu^{2+}, and AA. Concentrations and corresponding recovery percentages are listed in matching order. The results illustrate concentration-dependent variations in quantitative prediction accuracy, including substantial deviations at the lowest evaluated concentrations.

Analyte	Calibrated concentrations (µM)	Mean recovery at each concentration (%)
5-HT	0.05, 0.25, 1, 2.5	283, 110, 97, 88
DA	0.1, 0.25, 0.5, 1	149, 87, 94, 87
Cu^{2+}	0.05, 1, 2, 10	1240, 90, 70, 87
AA	2, 10, 30, 100	170, 71, 100, 90

Python code for FSCV data assemble using google colab

```
# Install needed packages (first time only)
!pip install openpyxl tqdm --quiet

import pandas as pd
from google.colab import files
from io import BytesIO
from tqdm import tqdm

# Step 1: Upload multiple files from your USB
print("Select all your .txt files from your USB drive")
uploaded = files.upload()  # You can select multiple files at once

# Step 2: Read each file and store as DataFrame
all_dfs = []

for fname in tqdm(uploaded.keys()):
    # Read two-column file (split by any whitespace/tab)
    df = pd.read_csv(BytesIO(uploaded[fname]), sep=r'\s+', header=None, engine='python')
    df = df.iloc[:, :2].copy()  # keep only first two columns
    df.columns = [f"{fname}_Col1", f"{fname}_Col2"]  # rename columns uniquely
    all_dfs.append(df)

# Step 3: Combine all files side-by-side
combined_df = pd.concat(all_dfs, axis=1)

# Step 4: Save as Excel
output_file = "combined.xlsx"
combined_df.to_excel(output_file, index=False)

# Step 5: Download the result
files.download(output_file)

print("✅ Done! Your Excel file is ready to download.")
```
